# Cross-Omics: Integrating Genomics with Metabolomics in Clinical Diagnostics

**DOI:** 10.3390/metabo10050206

**Published:** 2020-05-18

**Authors:** Marten H. P. M. Kerkhofs, Hanneke A. Haijes, A. Marcel Willemsen, Koen L. I. van Gassen, Maria van der Ham, Johan Gerrits, Monique G. M. de Sain-van der Velden, Hubertus C. M. T. Prinsen, Hanneke W. M. van Deutekom, Peter M. van Hasselt, Nanda M. Verhoeven-Duif, Judith J. M. Jans

**Affiliations:** 1 Section Metabolic Diagnostics, Department of Genetics, University Medical Centre Utrecht, Utrecht University, Lundlaan 6, 3584 EA Utrecht, The Netherlands; martenkerkhofs@gmail.com (M.H.P.M.K.); h.a.siepel-3@umcutrecht.nl (H.A.H.); a.m.willemsen.marcel@gmail.com (A.M.W.); M.vanderHam-3@umcutrecht.nl (M.v.d.H.); J.Gerrits@umcutrecht.nl (J.G.); M.G.deSain@umcutrecht.nl (M.G.M.d.S.-v.d.V.); B.Prinsen@umcutrecht.nl (H.C.M.T.P.); N.Verhoeven@umcutrecht.nl (N.M.V.-D.); 2 Section Metabolic Diseases, Department of Child Health, Wilhelmina Children’s Hospital, University Medical Centre Utrecht, Utrecht University, Lundlaan 6, 3584 EA Utrecht, The Netherlands; P.vanHasselt@umcutrecht.nl; 3 Section Genomic Diagnostics, Department of Genetics, University Medical Centre Utrecht, Utrecht University, Lundlaan 6, 3584 EA Utrecht, The Netherlands; K.L.I.vanGassen-2@umcutrecht.nl (K.L.I.v.G.); H.W.M.vanDeutekom-3@umcutrecht.nl (H.W.M.v.D.)

**Keywords:** cross-omics, untargeted metabolomics, genomics, diagnostics, data integration, next-generation sequencing, next-generation metabolic screening

## Abstract

Next-generation sequencing and next-generation metabolic screening are, independently, increasingly applied in clinical diagnostics of inborn errors of metabolism (IEM). Integrated into a single bioinformatic method, these two –omics technologies can potentially further improve the diagnostic yield for IEM. Here, we present cross-omics: a method that uses untargeted metabolomics results of patient’s dried blood spots (DBSs), indicated by Z-scores and mapped onto human metabolic pathways, to prioritize potentially affected genes. We demonstrate the optimization of three parameters: (1) maximum distance to the primary reaction of the affected protein, (2) an extension stringency threshold reflecting in how many reactions a metabolite can participate, to be able to extend the metabolite set associated with a certain gene, and (3) a biochemical stringency threshold reflecting paired Z-score thresholds for untargeted metabolomics results. Patients with known IEMs were included. We performed untargeted metabolomics on 168 DBSs of 97 patients with 46 different disease-causing genes, and we simulated their whole-exome sequencing results in silico. We showed that for accurate prioritization of disease-causing genes in IEM, it is essential to take into account not only the primary reaction of the affected protein but a larger network of potentially affected metabolites, multiple steps away from the primary reaction.

## 1. Introduction

Inborn errors of metabolism (IEMs) are monogenetic disorders defined as “any condition that leads to a disruption of a metabolic pathway” [1]. This definition includes enzyme or transporter deficiencies or superactivities, chaperone deficiencies, and deficiencies in transcription factors that can induce metabolic abnormalities [1]. After the discovery of the first IEM—alkaptonuria—in 1908, the number of known IEMs has risen exponentially. On 18 March 2020, the database IEMbase (https://iembase.org) that lists all IEMs included a total of 1442 known diseases [1]. With an estimated global birth prevalence of 1:2000, IEMs are responsible for a significant part of child morbidity and mortality [2].

Nowadays, -omics technologies, which provide a holistic view of large groups of biomolecules, are increasingly applied in clinical diagnostics of IEM. Next-generation sequencing (NGS) through whole-exome sequencing (WES) is of great value in this process, as approximately 85% of disease-causing mutations are located in coding and functional regions of the genome [3]. However, disease-gene discovery by WES is complicated by the large number of variants that are identified: over 80,000 variants in coding regions, of which 6000–10,000 are rare variants, in an individual exome [4,5]. Approximately one-third of these variants are predicted to result in nonsynonymous amino acid substitutions, small deletions or insertions, or alterations of splice sites. After common variants have been filtered out, variants deleteriously affecting protein function are identified based on inheritance pattern, prediction software taking into account conservation across species, population data, functional data, and phenotypic data [6]. Still, as an individual genome is estimated to harbor approximately 100 genuine loss-of-function variants, of which approximately 20 result in complete loss-of-function [7], strategies that complement NGS to identify the disease-causing gene are indispensable.

A second valuable -omics technique in the diagnostic process of identifying IEMs is metabolomics. This technology can characterize the metabolome of an organism in all kinds of body fluids, at a certain point in time under certain conditions. Using metabolomics technologies, small molecules can be identified, theoretically enabling the discovery of disturbances of all metabolic pathways present in the studied organism. If metabolomics studies are performed untargeted, a wide range of metabolites can be detected using only a single test in a hypothesis-free manner. Successful use of metabolomics approaches in clinical diagnostics of IEMs, referred to as next-generation metabolic screening (NGMS), has been reported by several groups [8,9,10,11,12,13,14]. Despite the potential of these approaches, independent confirmation of NGMS findings is still warranted.

Although the implementation of NGS as a diagnostic tool—and, to a far lesser extent, NGMS—have impacted clinical diagnostics of known and (yet) unknown IEMs, diagnostic yield and time-to-diagnosis may be further improved by integration of NGS and NGMS in a single bioinformatic method [15]. Here, we present such a method, which we called cross-omics, that uses untargeted metabolomics results of patient’s dried blood spots (DBSs) mapped onto human metabolic pathways to prioritize the patient’s genes that harbor potential pathogenic variants that result from WES.

We demonstrate the development of our cross-omics method and the optimization of three different parameters by performing untargeted metabolomics on DBSs from patients with known IEM, mapping these results onto human metabolic pathways, and by integrating this with in silico simulated WES results for each patient (i.e., 200 randomly selected protein-encoding genes of the human genome). We demonstrate that for accurate prioritization of disease-causing genes in IEMs, it is essential to take into account not only the primary reaction of the affected protein but a larger network of potentially affected metabolites, including metabolites multiple steps away from the primary reaction.

## 2. Results

During the development of the cross-omics method, we studied three parameters: (1) the maximum distance to the primary reaction of the affected protein (6 options); the extension stringency threshold reflecting in how many reactions a metabolite can participate, to be taken into account when extending the metabolite set associated with a certain gene (6 options); the biochemical stringency threshold reflecting paired Z-score thresholds for untargeted metabolomics results (4 options) (see Section 4 for methods).

### 2.1. Generation of Gene-Specific Metabolite Sets

**Figure 1:**
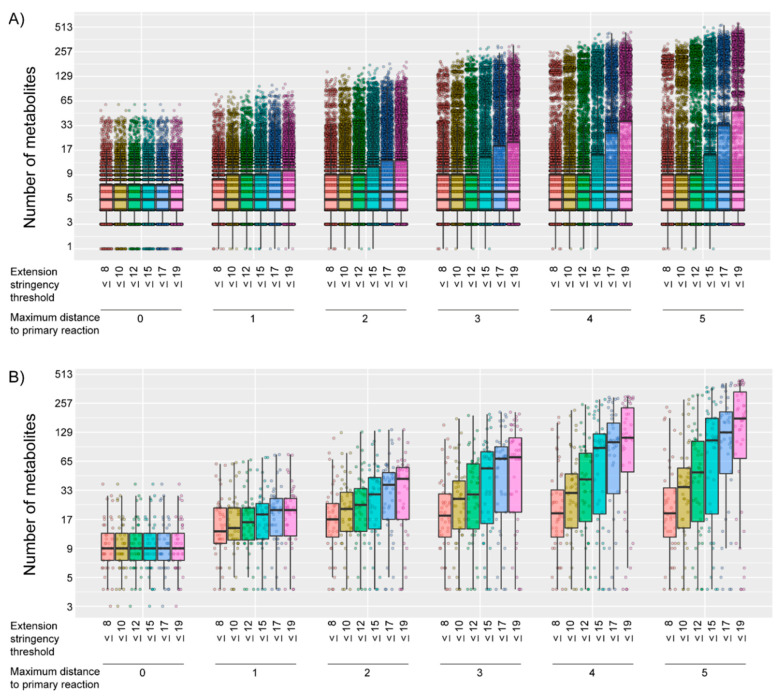
Number of metabolites on a logarithmic scale (log2 (“number of metabolites” + 1)) within the gene-specific metabolite set, for (**A**) all 7033 genes and (**B**) each of the 44 disease-causing genes with a gene-specific metabolite set that consisted of at least 1 metabolite in any parameter combination. The number of metabolites is stratified by the first studied parameter: maximum distance to the primary reaction and by the second studied parameter: the extension stringency threshold. Boxplots indicate the distribution of the number of metabolites in the gene-specific metabolite sets. Each dot represents one gene-specific metabolite set.

We were able to generate gene-specific metabolite sets, consisting of at least one Human Metabolome Database (HMDB) annotated metabolite, for 7033/18,974 of the protein-encoding genes. For these 7033 genes, the number of metabolites within the gene-specific metabolite set differed, depending on the combination of the parameters studied, with a median number of metabolites per gene-specific metabolite set of 5 or 6 (Figure 1A). As expected, the number of metabolites within a gene-specific metabolite set increased when the maximum distance to the primary reaction increased and/or when the extension stringency threshold was eased (Figure 1A). For some of the parameter combinations, the lowest number of metabolites within the gene-specific metabolite set was 0, while the highest number was 568 metabolites.

To determine, a priori, whether the 46 disease-causing genes of the included patients theoretically could be prioritized as potentially disease-causing, we checked whether the gene-specific metabolite sets of the patients’ disease-causing genes consisted of at least one metabolite. For two disease-causing genes, the gene-specific metabolite set consisted of zero metabolites, for all of the parameter settings. The gene-specific metabolite sets of all other disease-causing genes consisted of 1 to 443 metabolites, depending on the parameter combination (Figure 1B).

### 2.2. Assessment of the Most Favorable Parameter Combination for the Cross-Omics Method

We determined the most favorable combination of the 144 possible combinations (see Section 4 for methods) of the three assessed parameters by studying their effects on the diagnostic value of the cross-omics method. Per patient, gene prioritization was considered accurate when the patient’s disease-causing gene ranked within the top 10 of the prioritized list of 201 genes. The diagnostic value of the cross-omics method was defined as the fraction of correctly prioritized disease-causing genes. The cross-omics analysis was repeated 1000 times for all 97 included patients. The ten test sets consisted of 980 analyses, randomly selected from the 1000 analyses, and the ten validation sets consisted of the remaining 20 analyses (see Section 4 for methods).

**Figure 2:**
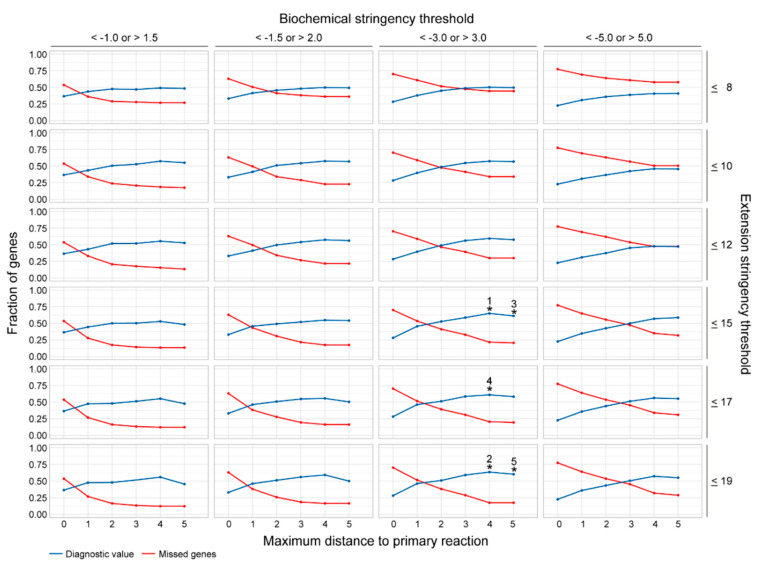
Assessment of the most favorable parameter combination for the cross-omics method. The diagnostic value of the cross-omics method for the different parameter combinations is depicted in blue, the fraction of missed disease-causing genes is depicted in red. The five parameter combinations reaching the highest diagnostic value are highlighted with numbered asterisks: 1: 0.65; 2: 0.64; 3: 0.61; 4: 0.61; 5: 0.60).

Out of all 95,060 cross-omics analyses in the ten test sets, the parameter combination with the highest diagnostic value consisted of a maximum distance to the primary reaction of 4, an extension stringency threshold of ≤15 and a biochemical stringency paired Z-score threshold of <−3.0 and >3.0 (Figure 2, Table 1). In general, the diagnostic value reached an optimum for each extension and biochemical stringency threshold when the maximum distance to the primary reaction increased to 4 or 5, in comparison to lower distances (Figure 2, Table 1). In addition, the fraction of missed disease-causing genes declined when the maximum distance to the primary reaction was increased up to 4. Likewise, the diagnostic value reached an optimum for an extension stringency threshold of >12, and a biochemical stringency threshold of <−3.0 or >3.0 (Figure 2).

In the 1940 analyses of the ten validation sets, nearly identical fractions of correctly prioritized disease genes were achieved for all parameter combinations as within the ten test sets, demonstrating the robustness of the most favorable parameter combination (Figure S1).

**Figure 3:**
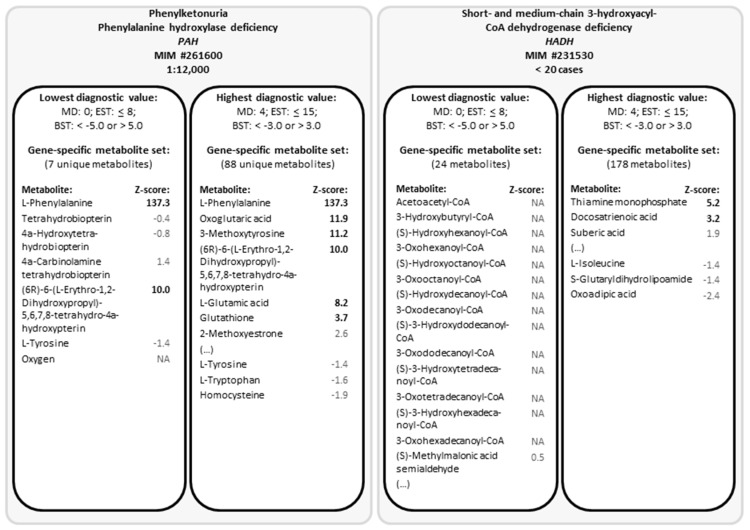
Gene-specific metabolite sets for phenylketonuria and short- and medium-chain 3-hydroxyacyl-CoA dehydrogenase deficiency for the parameter combination with the lowest and with the highest diagnostic value. Abbreviations: BST, biochemical stringency threshold; EST, extension stringency threshold; MD, maximum distance to the primary reaction; MIM, (online) Mendelian Inheritance in Man (number); NA, not assessed.

In Figure 3, we demonstrated for two IEMs which metabolites the gene-specific metabolite sets contained, for the most favorable parameter combination (with the highest diagnostic value) as well as for the parameter combination with the lowest diagnostic value (Figure 3). Hereby, we illustrate which metabolites contribute to the prioritization of the affected gene. For phenylketonuria, which is due to phenylalanine hydroxylase deficiency, the gene-specific metabolite set with the parameter combination resulting in the lowest diagnostic value, contained seven metabolites including l-phenylalanine, which has an impressive Z-score. Due to the Z-score of l-phenylalanine, PAH was always prioritized correctly, independent of the parameter combination. In contrast, the parameter combination resulting in the lowest diagnostic value resulted in a gene-specific metabolite set for short- and medium-chain 3-hydroxyacyl-CoA dehydrogenase deficiency of 24 metabolites, of which none had a Z-score above or below the biochemical stringency threshold. This is mainly because most metabolites in the primary metabolite contain a –CoA group, which is highly reactive and is therefore not measured using direct-infusion metabolomics. However, using the parameter combination resulting in the highest diagnostic value, the gene-specific metabolite set contained 178 metabolites, of which two had a Z-score above the biochemical stringency threshold, resulting in correct prioritization.

### 2.3. Assessment of the Robustness of the Cross-Omics Method

**Figure 4:**
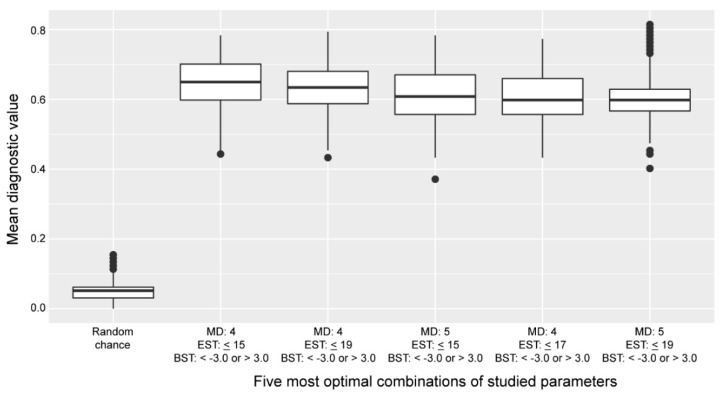
Distribution of the diagnostic values of the five most favorable parameter combinations of the cross-omics method compared to the diagnostic value if based on random chance. Boxplots indicate the distribution of the diagnostic values over 1000 cross-omics analyses. Abbreviations: BST, biochemical stringency threshold; EST, extension stringency threshold; MD, maximum distance to the primary reaction.

The diagnostic value of the cross-omics method, if based on random chance, was calculated to be 0.05 (see Section 4 for methods). All five most-favorable parameter combinations scored better than random chance (Figure 4).

**Figure 5:**
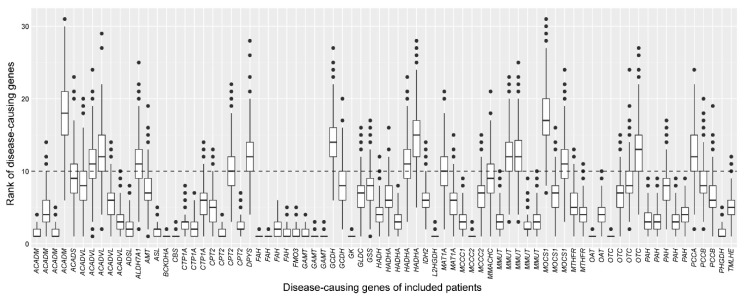
Robustness of the cross-omics method on a within-patient basis. The consistency of the ranks of the disease-causing genes across 1000 analyses is demonstrated by boxplots, for the most favorable parameter combination: maximum distance to primary reaction = 4, extension stringency threshold <15, biochemical stringency paired Z-score threshold is <−3.0 or >3.0. Disease-causing genes of individual patients are depicted on the *x*-axis; the ranks of the disease-causing genes are depicted on the *y*-axis. The horizontal dashed line at y = 10 depicts the maximum rank of the disease-causing gene that was considered to be prioritized correctly. All 21 patients whose disease-causing genes were missed were omitted from this figure.

To investigate the robustness of the cross-omics method on a within-patient basis, we visualized the distribution of the ranks of the patients’ disease-causing genes per patient for the most favorable parameter combination (Figure 5). Using this combination, on average, 61 disease-causing genes were correctly prioritized, with 35 disease-causing genes always correctly prioritized—for example, one of these being *PAH* (Figure 3). The maximum difference between the ranks of the disease-causing gene within a single patient was 27 (rank 2–rank 29, *ACADVL*), due to the scores of other genes that were present in the simulated WES results. Twenty-one patients’ disease-causing genes were always missed, since their gene-specific metabolite sets hardly contained any metabolite that was identified using direct-infusion untargeted metabolomics, impeding enrichment of the set and thereby precluding correct prioritization.

## 3. Discussion

Technological advancements in –omics technologies are of substantial value for clinical diagnostics of IEMs. Implementation of NGS as a first-tier diagnostic tool has greatly increased the diagnostic yield and reduced diagnostic delay, which are both important benefits for patients [16,17,18]. Although the implementation of NGMS in clinical diagnostics is in its infancy when compared to NGS, several recent studies reported its potential applicability in clinical diagnostics of IEMs for individual patients [8,9,10,11,12,13,14]. Notwithstanding its added value, NGMS is—unlike NGS—not expected to become a stand-alone diagnostic tool, as genetic confirmation of the resulting diagnosis is often required. On the other hand, despite the relatively high diagnostic yield of NGS in several studies, NGS does not provide a definite diagnosis for a substantial number of patients [19,20]. Variants of uncertain significance (VUS) in known disease genes or potentially deleterious variants in genes with uncertain clinical significance may be identified and the list of genes with VUS is often long. Functional studies such as NGMS may provide support for the (in) significance of a given variant. As such, NGMS may uncover abnormalities that can be linked to a genetic variant, thereby providing powerful evidence for the pathogenicity of the variant [21]. Therefore, it is expected that NGMS may aid gene prioritization upon NGS when no clear genetic diagnosis can be made and many genes harbor VUS.

Ideally, data from NGS and NGMS platforms are integrated to achieve the most complete and accurate diagnostic output. However, taking full benefit of all data by meaningfully coupling the output of these multiple –omics platforms remains a significant challenge. Whereas associating metabolomes with genomes on population scale is already challenging, integrating –omics data for individual patients comes with additional challenges. In addition to the many layers of complexity in the biological regulation of genetically controlled metabolite levels, the human metabolome is also strongly affected by other factors such as nutrition, exercise, and the gut microbiome.

Here, we demonstrate the development of a bioinformatic method—termed cross-omics—that couples untargeted metabolomics results to in silico simulated WES results, indicated by gene names. We studied three parameters which are important for accurately prioritizing potentially disease-causing genes. The first parameter was the maximum distance to the reaction of the affected protein, the primary reaction. Since metabolites upstream or downstream (before or after the metabolic block) may also be affected, the diagnostic value may increase when a larger metabolic network is taken into account. Tyrosinaemia type I, for example, results from a deficiency of fumarylacetoacetase (gene: *FAH*). Typical biochemical findings include elevated concentrations of plasma tyrosine, even though this metabolite is four steps upstream of the primary enzymatic defect. We demonstrated that, indeed, taking into account metabolites up to four reactions away from the primary reaction resulted in the most favorable diagnostic value of the cross-omics method, which we also illustrated for *HADH* (Figure 3). Though, for some disease-causing genes, such as *PAH*, including only the primary reaction would be already sufficient for correct prioritization of the gene (Figure 3).

The five most favorable extension stringency thresholds were all >12 (≤15, ≤17, ≤19). This means that the extension of gene-specific metabolite sets should be based on all metabolites that participate in up to 15 reactions. The diagnostic value of the cross-omics method was considerably lower if the extension of gene-specific metabolite sets was discontinued via metabolites that participated in a smaller number of reactions. The most favorable biochemical stringency of the paired Z-score threshold turned out to be <−3.0 and >3.0. Interestingly, this paired Z-score threshold is more stringent than often used for NGMS [8,11,12,14], although for most diagnostic metabolites in IEMs, Z-scores are >3.0 [8,10,11,12,13,14]. As is true for the maximum distance to the primary reaction, the most favorable paired Z-score threshold may also vary for the different IEMs [14], as some IEMs may present with more subtle metabolic aberrations than others [8,10,11,12,13,14].

An important limitation of the cross-omics method is that genes for which no gene-specific metabolite set can be generated, cannot be taken into account, even though these genes might cause IEMs. We could generate gene-specific metabolite sets for 7033 of the 18,974 protein-encoding genes, but for the majority of the protein-encoding genome, no gene-specific metabolite set could be generated. As expected, this is mostly due to the fact that these genes do not encode proteins expected to affect metabolite levels, but database annotation may also be incomplete, additional gene-protein-metabolite links are yet to be uncovered, and metabolites might not be identified using direct-infusion metabolomics. This limitation precluded the correct prioritization of the affected genes of 21 patients included in this study. Also, as samples of untreated patients are very scarce, samples of patients under clinical management were included, which resulted in the inclusion of four samples wherein the known biomarkers of the patient’s IEM were completely normalized. These four samples slightly decreased the diagnostic value of the cross-omics method. Moreover, in other samples of patients under clinical management, the extent of the aberrations might have been reduced in comparison to untreated samples. Therefore, we speculate that the diagnostic value in samples of undiagnosed—and thus untreated—patients might be higher than reported here.

Still, the diagnostic value of the cross-omics method is currently still insufficient for diagnostic use in clinical practice. For further optimization of the method, patients with a wide range of genetic diseases should be included for whom WES results are available. Moreover, next to the small-molecule, hydrophilic metabolites that are presently taken into account, the cross-omics method might be further optimized by including other –omics platforms, such as lipidomics, glycomics, or even transcriptomics. Moreover, in addition to the optimization of the three parameters that we discussed, many more parameters—such as centrality metrics, as utilized in network science—could influence the diagnostic value of the cross-omics method. It requires further study of whether other parameters result in the improvement of the diagnostic value.

For implementation into clinical practice, cross-omics requires the availability of detailed WES results, also including the gene names of genes that harbor VUS, intronic variants, and copy-number variants. In many laboratories, generating such a list of gene names is not standard practice. To improve the implementation of cross-omics, we envision that cross-omics could also be performed the other way around: a gene candidate list is generated based on results of untargeted metabolomics analyses, and this generated gene panel is studied using NGS.

A major strength of the current cross-omics method is its platform-independent nature, as the input parameters are rather straightforward: the biochemical input only consists of metabolite Z-scores, which can be calculated for any dataset regardless of the untargeted metabolomics approach that is used [14]. Likewise, the genetic input consists of gene names, so that both WES and WGS results, independent of the sequencing characteristics, may serve as genetic input. This platform-independent nature ensures that any metabolic diagnostic laboratory can test, use, and improve the cross-omics method.

## 4. Materials and Methods

### 4.1. Sample Inclusion

IEMs of interest for this study were IEMs with one or more known small-molecule biomarkers in blood, such as urea cycle disorders, organic acidurias, amino acid metabolism disorders, and fatty acid oxidation disorders. Patients genetically or enzymatically confirmed to be affected with such IEM were included when one or more remnant DBSs was available in the metabolic diagnostic laboratory of the University Medical Centre Utrecht.

We included 168 DBSs of 97 unique patients. Each patient had either a known disease-causing gene (92 patients, all homozygous or hemizygous pathogenic or likely pathogenic mutations) or a known enzymatic defect but without genetic confirmation (5 patients). For these latter 5 patients we selected, the gene most commonly causing the disease according to the Online Mendelian Inheritance in Man (OMIM) database for further analyses [22]. Together, the 97 unique patients represented 46 unique disease-causing genes, all representing IEMs with one or more known small-molecule biomarkers in blood. Thirty DBS samples of individuals in whom an IEM was excluded after a thorough routine diagnostic work-up were included as control samples.

Patient samples were included based on availability and not based on whether a patient was under clinical management at the time of sampling or not, nor based on the presence of abnormal biochemical findings. Although the biochemical profile remained distinct in almost all samples, in four samples, the known biomarkers of the patient’s IEM were normalized due to treatment [11].

All included patients, or their legal guardians, approved the possible use of their remaining samples for method validation, in agreement with institutional and national legislation. All procedures followed were in accordance with the ethical standards of the University Medical Centre Utrecht and with the Helsinki Declaration of 1975, as revised in 2000.

### 4.2. Cross-Omics Method

**Figure 6:**
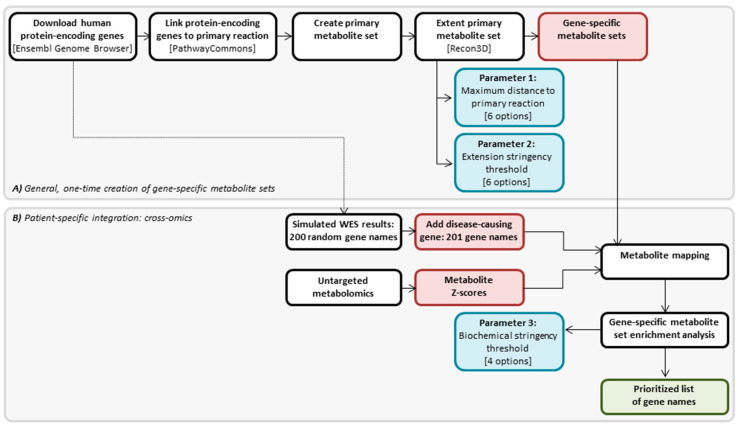
Cross-omics method. (**A**) General, one-time creation of gene-specific metabolite sets. (**B**) Patient-specific integration: cross-omics. Different steps in the cross-omics method are depicted in white boxes, three studied parameters during the development of the cross-omics method are depicted in blue boxes, the input for the metabolite mapping step is depicted in red boxes, and the product of the cross-omics method is depicted in the green box.

The cross-omics method that we designed consists of two parts: (A) one-time generation of gene-specific metabolite sets, for each human protein-encoding gene, and (B) a patient-specific part that integrates metabolite Z-scores resulting from untargeted metabolomics and in silico simulated WES results reflected by gene names, into a prioritized gene list. An overview of the cross-omics method is presented in Figure 6, and both parts of the method are further explained in the following sections. The cross-omics method was written in R programming language [23].

In the development of the cross-omics method, we studied the effect on the diagnostic value of the method of three different parameters: in Part A, the extension of the metabolite set, including (1) the maximum distance of the included metabolites in the set to the primary reaction of the encoded protein and (2) the extension stringency, i.e., a threshold for the number of reactions in which a metabolite can participate before a further extension of the gene-specific metabolite set is discontinued; in part B, (3) the biochemical stringency, i.e., paired Z-score thresholds for the degree to which metabolites are increased or decreased to be considered significantly altered (Figure 6). All three studied parameters are further explained in the following sections.

### 4.3. Cross-Omics Method Part A: One-Time Generation of Gene-Specific Metabolite Sets

A list of 65,071 human genes was downloaded from the Ensembl Genome Browser [24] (Figure 6). Genes were selected when they were protein-encoding, and when they had a valid and unique HUGO Gene Nomenclature Committee gene name. This resulted in 18,974 genes for which, theoretically, metabolite sets could be created.

For each of the 18,974 genes, the encoded protein was coupled to its primary reaction (i.e., the enzymatic reaction catalyzed by the protein, or the transporter function of the protein) using the R package PaxtoolsR and the biological pathway and interaction database PathwayCommons [25] (Figure 6). Substrates and products of the primary reaction together formed the primary metabolite set. This primary metabolite set was defined as 0 steps from the primary reaction.

**Figure 7:**
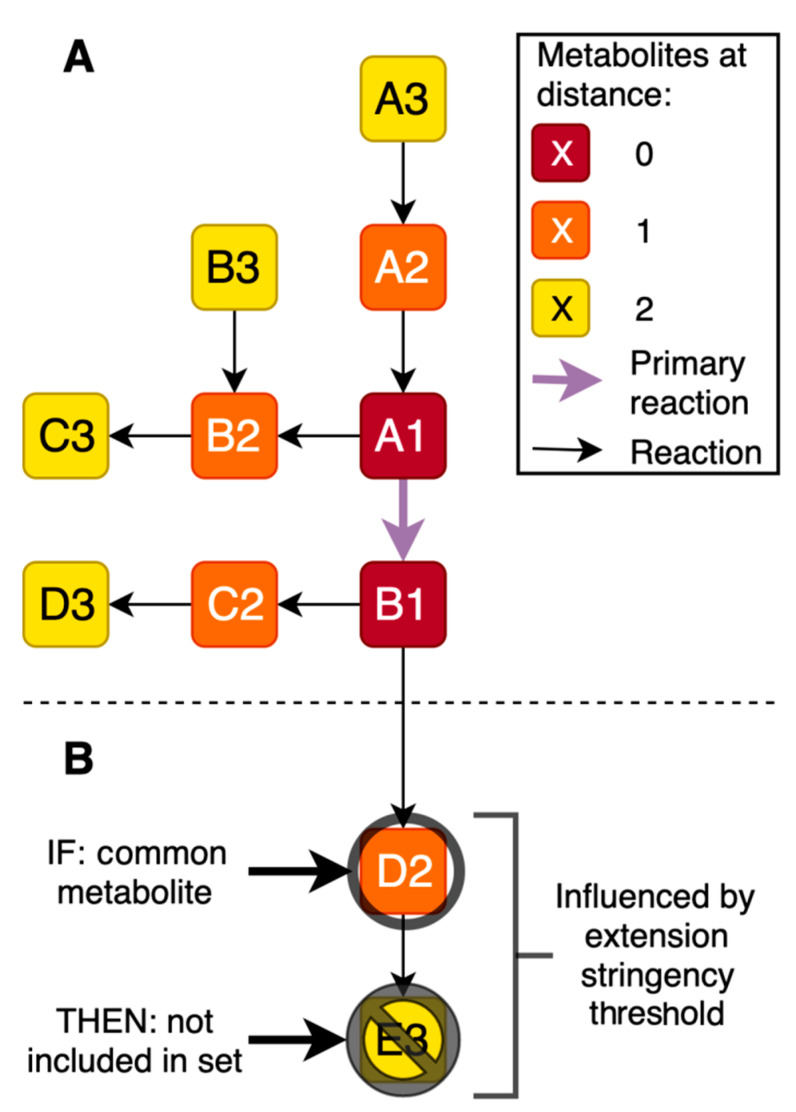
Metabolite set extension. (**A**,**B**) Reactions are depicted by black arrows, metabolites are depicted by lettered boxes. Reactions associated with metabolites are used to identify metabolites one or more steps away from the primary reaction. The maximum distance is the first studied parameter during the development of the cross-omics method. (**B**) Extension stringency threshold: e.g., if metabolite D2 is present in more reactions than the defined threshold, metabolite E3 is not included in the gene-specific metabolite set via metabolite D2. The extension stringency threshold is the second studied parameter during the development of the cross-omics method.

Next, each gene-specific primary metabolite set was extended to investigate whether including more distant metabolites had added value. For each metabolite in the primary set, all known reactions in which the metabolites participate were queried in the metabolite-reaction database Recon3D [26] (Figure 6 and Figure 7). All these metabolites were added to the respective metabolite sets, forming the secondary gene-specific metabolite sets. This metabolite extension step is the first parameter that we studied in detail during the development of the cross-omics method, aiming to define the maximum distance of the included metabolites in the set to the primary reaction of the encoded protein. To this end, the extension of the gene-specific metabolite sets was repeated up to 5 times, with the respective metabolites of each iteration 1 to 5 steps (i.e., reactions) away from the primary reaction, resulting in six different options for the first studied parameter.

Non-specific metabolites that are products or substrates in many different reactions and many different metabolic pathways extend the gene-specific metabolite sets to containing almost all known metabolites and, thus, loss of specificity. To avoid inclusion of these non-specific metabolites, two filtering steps were conducted. First, twenty non-specific metabolites such as H_2_O and H^+^ (listed in Table S1) were removed before any extensions or calculations were performed. Second, we studied a second parameter during the development of the cross-omics method: the extension stringency, i.e., a threshold for the number of reactions in which a metabolite can participate before a further extension of the gene-specific metabolite set should be discontinued (Figure 7). When a metabolite met the threshold, further extension of the gene-specific metabolite set via this metabolite was not performed, although the metabolite itself remained in the set. We studied extension stringency thresholds of ≤8, ≤10, ≤12, ≤15, ≤17, or ≤19 reactions in which a metabolite participated, resulting in six different options for the second studied parameter.

### 4.4. Cross-Omics Method Part B: Patient-Specific Integration: Cross-Omics

#### 4.4.1. In Silico Simulation of WES Results

As in most patients, no WES was performed during the diagnostic process, and since ethically, WES could also not be performed for the purpose of this study, WES results were simulated in silico for each patient. Next to the pathogenic mutation(s) in the disease-causing gene, patients will harbor variants (e.g., likely pathogenic variants or variants of unknown significance) in many more genes. After filtering, in patients where non-consanguineous parent variants are detected in approximately 10 genes, in consanguineous families in approximately 50 genes, and, when silent and intronic variants are included, variants are detected in approximately 100 genes. In order to preclude underestimation of the magnitude of patients’ WES results, we decided to randomly select 200 genes from the 18,974 selected protein-encoding genes (methods, Section 4.3) for each patient. After the addition of the patient’s disease-causing gene, in silico simulated WES results of each patient included 201 genes, indicated by gene names (Figure 6).

#### 4.4.2. Direct-Infusion High-Resolution Mass Spectrometry

Sample collection, sample preparation, and metabolomics analysis were performed as previously described [11]. In short, untargeted metabolomics was performed by direct-infusion high-resolution mass spectrometry, using a TriVersa NanoMate system (Advion, Ithaca, NY, USA) controlled by Chipsoft software (version 8.3.3, Advion, Ithaca, NY, USA), mounted onto the interface of a Q-Exactive high-resolution mass spectrometer (Thermo Scientific™, Bremen, Germany). For each sample, technical triplicates were analyzed, infusing each sample three times into the mass spectrometer. Scan range was 70 to 600 *m*/*z.*

Mass peak annotation was performed by matching the *m*/*z* value of the mass peak with a range of two parts per million to monoisotopic metabolite masses present in the HMDB, version 3.6 [27]. Annotations of metabolites that can occur endogenously were selected: >1800 unique *m*/*z* per batch, corresponding to >3800 metabolite identifications, in line with previous analyses [11]. For each mass peak per patient sample, the deviation from the intensities in the control samples was indicated by a Z-score, calculated by Z-score = (intensity patient sample − mean intensity control samples)/standard deviation intensity control samples. Z-scores were calculated for both patient and control samples (Figure 6).

#### 4.4.3. Metabolite Mapping

The integration of simulated WES results and untargeted metabolomics results of each patient (i.e., the actual cross-omics) was performed by mapping patient’s metabolite Z-scores onto the gene-specific metabolite set of each gene in the patient’s simulated WES results (Figure 6). If for a patient, multiple DBSs were analyzed, metabolite Z-scores were averaged. Isomers cannot be distinguished in direct-infusion based metabolomics, and, therefore, isomers were considered a single entity with multiple HMDB annotations in both metabolite Z-scores and gene-specific metabolite sets.

#### 4.4.4. Gene-Specific Metabolite Set Enrichment Analysis

For each gene in the patient’s simulated WES results, the number of metabolites within the gene-specific metabolite set that exceeded a positive or negative Z-score threshold was calculated. This biochemical stringency threshold is the third parameter studied during the development of the cross-omics method. We studied four paired thresholds: (1) Z-scores <−1.0 or >1.5; (2) Z-scores <−1.5 or >2.0; (3) Z-scores <−3.0 or >3.0 and (4) Z-scores <−5.0 or >5.0.

Next, the number of metabolites exceeding the biochemical stringency threshold was used in a Fisher’s exact test, which assessed the significance of the enrichment of the patient’s gene-specific metabolite sets (Figure 6). Subsequently, all 201 genes from the simulated WES results were ranked on *p*-values of the Fisher’s exact test, with the lowest *p*-value ranked first. Hereby, the result of the cross-omics method is—per patient—a prioritized list of 201 genes (Figure 6).

For genes that lack a gene-specific metabolite set, or that lack any aberrant metabolites, a Fisher’s exact test could not be performed. These genes were defined as “missed”, and were assigned a *p*-value of 1.0.

### 4.5. Assessment of the Most Favorable Parameter Combination for the Cross-Omics Method

To determine the most favorable combination of the 144 possible combinations of the three assessed parameters (maximum distance to the primary reaction: six options; extension stringency threshold: six options; biochemical stringency threshold: four options), we studied their effects on the diagnostic value of the cross-omics method. Per patient, gene prioritization was considered accurate if the patient’s disease-causing gene ranked within the top 10 of the prioritized list of 201 genes. The diagnostic value of the cross-omics method was defined as the fraction of correctly prioritized disease-causing genes.

### 4.6. Assessment of the Robustness of the Cross-Omics Method

After the most favorable combination of parameters was identified, we studied its robustness. Hereto, we repeated the cross-omics analysis 1000 times, each time with a unique set of 200 randomly selected genes in the patient’s simulated WES results. Of these 1000 analyses, ten test- and validation sets were generated. The test sets consisted of 980 analyses, randomly selected from the 1000 analyses, and the validation sets consisted of the remaining 20 analyses.

To demonstrate that the cross-omics method outperforms random chance, we compared the diagnostic values of the cross-omics method using the most favorable parameter combinations to the diagnostic value that is expected based on random chance. Prioritization of disease-causing genes by random chance was tested as follows: we created a simulated disease-causing gene by randomly assigning a number between 1 and 201; we created a simulated top 10 of prioritized genes by randomly selecting 10 numbers between 1 and 201; we determined the occurrence of the simulated disease-causing gene in the simulated top 10; we repeated this simulation 1000 times for 97 simulated disease-causing genes, and we calculated the fraction of correct prioritization within the top 10.

### 4.7. Data Availability

The untargeted metabolomics method has been described here [11], and the R code of the untargeted metabolomics method is available online [28]. Data used for the cross-omics method are available upon request, and R code is available online [29].

## 5. Conclusions

In conclusion, we here present the development of our platform-independent cross-omics method, which integrates untargeted metabolomics results and WES results, providing a prioritized list of potentially disease-causing genes. We have demonstrated that for accurate prioritization of disease-causing genes in IEMs, it is essential to take into account not only metabolites involved in the primary metabolic reaction of the affected protein but a larger network of potentially affected metabolites, including metabolites up to four steps away from the primary metabolic reaction.

## Figures and Tables

**Table 1:** Most favorable parameter combinations for the cross-omics method.

Rank	Diagnostic Value	Missed Fraction	Maximum Distance	Extension Stringency	Biochemical Stringency
1	0.65	0.22	4	≤15	<−3.0; >3.0
2	0.64	0.18	4	≤19	<−3.0; >3.0
3	0.61	0.21	5	≤15	<−3.0; >3.0
4	0.61	0.21	4	≤17	<−3.0; >3.0
5	0.60	0.18	5	≤19	<−3.0; >3.0
6	0.59	0.30	4	≤12	<−3.0; >3.0
7	0.59	0.17	4	≤19	<−1.5; >2.0
8	0.59	0.29	3	≤19	<−3.0; >3.0
9	0.59	0.33	3	≤15	<−3.0; >3.0
10	0.59	0.32	5	≤15	<−5.0; >5.0

## Supplementary Materials

The following are available online at https://www.mdpi.com/2218-1989/10/5/206/s1, Figure S1: Assessment of the most favorable parameter combination of the cross-omics method in the ten test and validation sets. Table S1: Twenty non-specific metabolites removed before metabolite set extension.
