# Supplementary material for: Cross-Omics: Integrating Genomics with Metabolomics in Clinical Diagnostics
Source: Metabolites. 2020 May 18;10(5):206. doi: 10.3390/metabo10050206 (PMC7281020; doi:10.3390/metabo10050206)
Supplement: Supplementary file 1 [file metabolites-10-00206-s001.pdf]

**Table S1.** Twenty non-specific metabolites removed before metabolite set extension.

| Metabolite | ChEBI | KEGG | PubChem |
|---|---|---|---|
| $H_2O$ | 15377 | C00001 | 962 |
| $O_2$ | 15379 | C00007 | 977 |
| $H^+$ | 15378 | C00282 | 1038 |
| $CO_2$ | 16526 | C00011 | 280 |
| $HPO_4^{2-}$ | 43474 | C00009 | 1004 |
| ATP | 15422 | C00002 | 5957 |
| dATP | 16284 | C00131 | 15993 |
| ADP | 16761 | C00008 | 6022 |
| dADP | 16174 | C00206 | 188966 |
| AMP | 16027 | C00020 | 6083 |
| dAMP | 17713 | C00360 | 12599 |
| Diphosphate | 29888 | C00013 | 1023 |
| Nicotinamide adenine dinucleotide phosphate - reduced | 57783 | C00005 | 22833512 |
| Nicotinamide adenine dinucleotide phosphate | 18009 | C00006 | 5886 |
| Nicotinamide adenine dinucleotide - reduced | 16908 | C00004 | 439153 |
| Nicotinamide adenine dinucleotide | 15846 | C00003 | 5893 |
| Flavin adenine dinucleotide oxidized | 16238 | C00016 | 643975 |
| Flavin adenine dinucleotide reduced | 17877 | C01352 | 446013 |
| Coenzyme A | 15346 | C00010 | 6816 |
| Hydrogen peroxide | 16240 | C00027 | 784 |

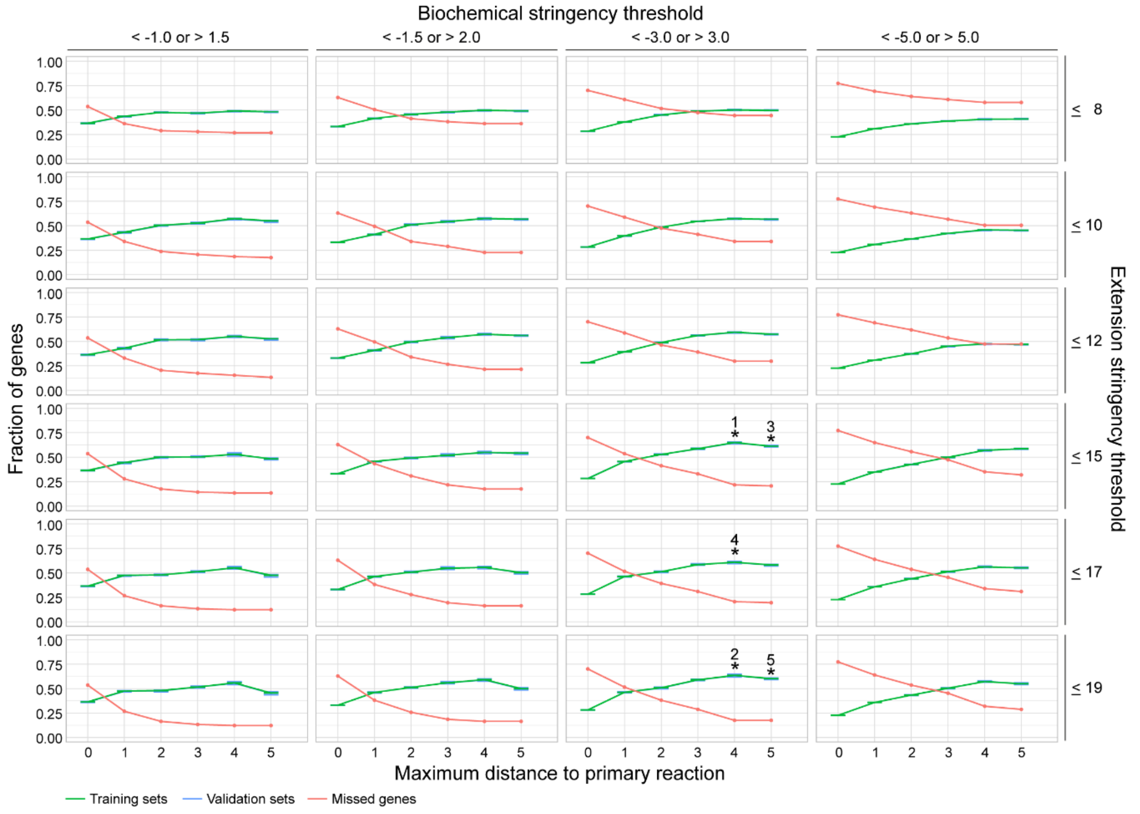


**Figure S1.** Assessment of the most favorable parameter combination of the cross-omics method in the ten test and validation sets. The diagnostic value of the cross-omics method for the different parameter combinations in the validation sets is depicted in blue, the diagnostic value in the test sets is depicted in green (overlaying the blue lines), and the fraction of missed disease-causing genes is depicted in red. The five parameter combinations reaching the highest diagnostic value are highlighted with numbered asterisks: 1: 0.65; 2: 0.64; 3: 0.61; 4: 0.61; 5: 0.60).
